# Crohn's Disease Presenting with Pyogenic Liver Abscess: A Case Report

**DOI:** 10.1155/2012/762480

**Published:** 2012-08-08

**Authors:** Sharon McGreal, Rupert Sayers, Peter Wurm, Kevin West

**Affiliations:** ^1^Department of Digestive Diseases, Leicester Royal Infirmary, Leicester GP ST2, UK; ^2^Department of Pathology, Leicester Royal Infirmary, Leicester GP ST2, UK

## Abstract

Pyogenic liver abscess (PLA) is a rare extraintestinal complication of Crohn's disease (CD), and the clinical and laboratory findings may emulate the reactivation of CD, therefore, delaying diagnosis. In this paper the patient presented with PLA as the initial manifestation of CD and experienced severe disease. The finding of PLA was established by computed tomography and initial treatment involved percutaneous drainage and antibiotics. The diagnosis of CD was made after colonoscopy and histological investigations.

## 1. Introduction

CD is a chronic inflammatory disease that is characterised pathologically by chronic mucosal inflammation and noncaseating granulomas. This process most frequently affects the terminal ileum but may involve any part of the gastrointestinal tract. The affected area shows a transmural pattern of inflammation which may be in discontinuity demonstrating skip lesions.

CD is a systemic illness that can present with multiple extraintestinal manifestations including hepatobiliary complications. Other complications such as cholelithiasis, primary sclerosing cholangitis, and pericholangitis are well documented; however, PLA is rare.

PLA usually develops secondary to biliary infections or infections of those organs drained by the portal vein appendicitis or diverticulitis. Despite the fact that portal pyemia is frequently found in IBD, the development of PLA remains rare, and most cases described to date have been in patients with established CD [1]. PLA as the initial manifestation of CD is even rarer. We present a patient in whom a complex multiloculated liver abscess was the initial manifestation of CD. 

## 2. Case Presentation

An eighteen-year-old male presented with a thirteen-day history of lethargy, poor appetite, and frequent, nonbloody diarrhoea. Over this period, he experienced drenching night sweats, rapid weight loss, bone pain, and myalgia. Prior to this he had been healthy with no significant family history or prescription medications.

On physical examination, he was clinically anaemic, cachectic, and dehydrated with a sinus tachycardia of 120 beats per minute and temperature of 40°C; examination of his abdomen showed some tenderness in the right upper quadrant. 

Laboratory results revealed a white blood cell count of 19.3 10^9^/L with a predominant neutrophilia and a microcytic anaemia. Other laboratory results showed a CRP of 195 mg/L and alkaline phosphate of 292 IU/L (see Table 1).

An unprepared sigmoidoscopy revealed a normal distal colon to the level of the splenic flexure. Further examination was not possible in view of faecal residue. A computerised tomography was requested to look into the possibility of more proximal CD or intraabdominal collection and identified a complex multiloculated, noncommunicating liver abscess. In addition, there was thickening of the terminal ileum up to the ileocaecal valve. 

The patient underwent percutaneous drainage of the abscess, draining 150 mLs of frank yellow pus from the largest locule. Over a one-week period, two further locules were drained with a total of 700 mLs of pus. The patient received two units of packed cells and was commenced on intravenous Imipenem.

Twenty four hours after drain insertion, the patient had become apyrexial and after 48 hours the white cell count had dropped to 9.3 × 10^9^/L (4−11 × 10^9^). The abscess culture grew streptococcus milleri on all samples. The patient was discharged from hospital thirteen days after his admission.

Four weeks after discharge, a colonoscopy was performed (see Figure 2). On endoscopy the entire colon looked normal but intubation of the terminal ileum revealed active mucosal inflammation with pleomorphic ulceration. The biopsies from the ileocaecal region showed some architectural distortion in association with patchy active chronic inflammation. The remainder of the large bowel showed no significant histological abnormality. These features were regarded as highly suggestive of CD.

Despite initial good progress, sixteen months later, his symptoms worsened and a subsequent MRI showed active CD in segments of the distal ileum and a low-grade partial bowel obstruction with a possible enteroenteric fistula. A repeat colonoscopy demonstrated active ulceration of the terminal ileum involving the ileocoecal valve with histology showing distortion and inflammation consistent with active CD. Two months later, he was admitted with abdominal pain and underwent a laparotomy revealing enteroenteric fistula and abscess formation. An extended right hemi colectomy and end ileostomy was formed. He underwent re-anastomosis following a symptom free period with no signs of intra abdominal abscess.

Routine endoscopic evaluation of the anastomosis six months later showed reactivated disease at the anastomotic site and patchy inflammation of the transverse colon. Immunosuppressive treatment with Azathioprine was commenced. The patient is currently well but had a hospital admission with renal calculi.

## 3. Discussion

**Figure 1:**
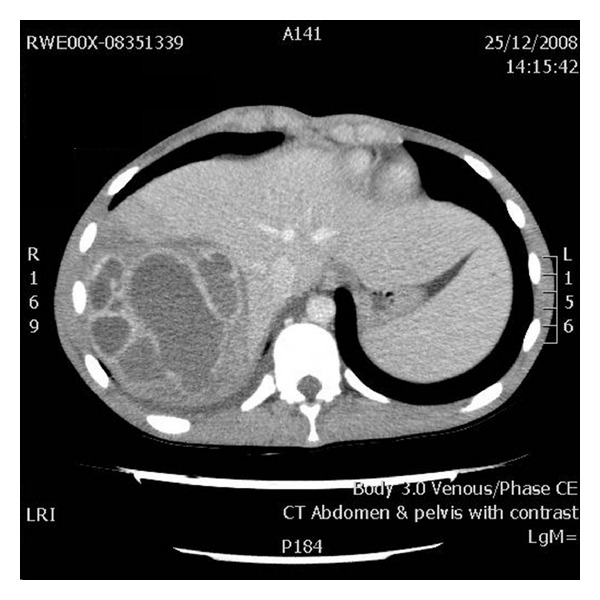
CT scan demonstrating multiloculated liver abscess containing at least 10 locules.

**Figure 2:**
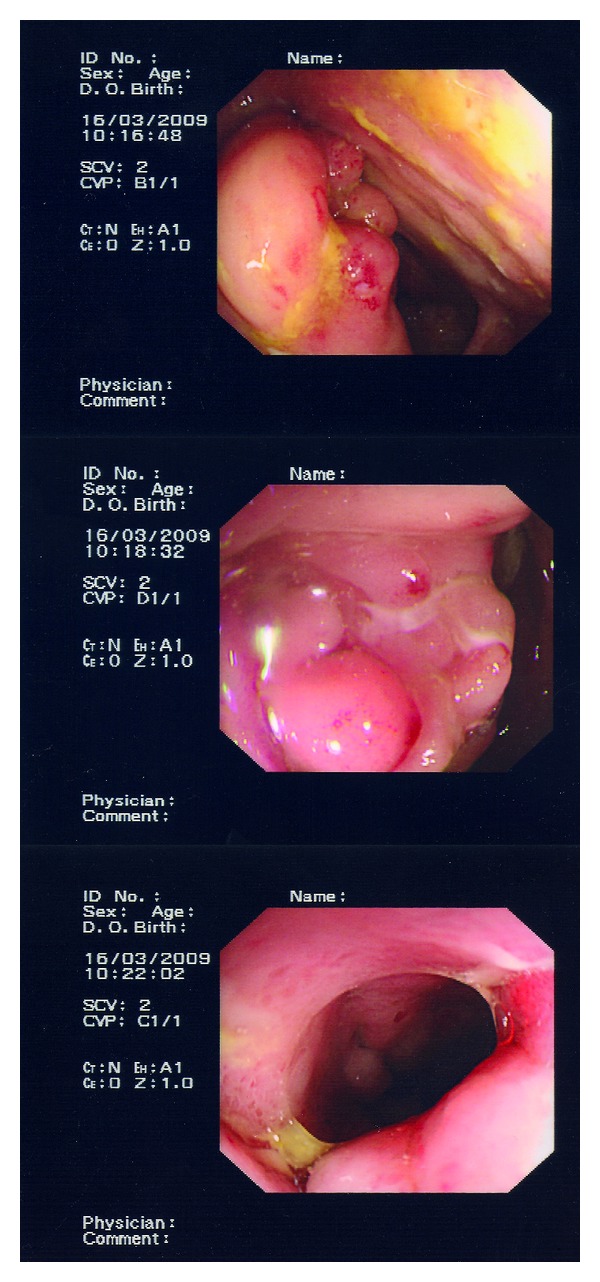
Colonoscopy images. Top: ilealcaecal valve ulceration, bottom: terminal ileum ulceration.

CD presenting with PLA is rare, [2] but the mortality has been reported to be high if diagnosis or treatment is delayed [3]. In this case, the diagnosis was made promptly through CT scan and percutaneous drainage performed thereafter, however, the course of disease has been severe (see Figure 1).

The incidence of LA in patients with CD is estimated at 10–15 times that found in the general population [1]. The average age of development has been quoted as 24–44 years [1] and in most cases the LA has occurred in well-established CD. There have been over 30 cases of reported LA complicating CD, however, only seven have presented as the initial manifestation of CD [4]. In cases reporting LA, a male predominance has been supported with a male to female ratio of 23 : 8; this is in contrast to the equal male to female ratio found in CD in general.

A number of factors are thought to predispose the formation of LA in CD. These include intraabdominal abscess, fistula formation, as well as mucosal ulceration, perforating disease, malnutrition, high-dose steroid, and antibiotic therapy, especially metronidazole. 

The mechanisms of spread remain elusive and several theories have been documented. These include direct extension of an intraabdominal abscess into the liver via the portal vein from an intraabdominal infection, and indirectly from complications of CD (i.e., biliary disease). In this case, it is possible that inflammation of ileum and ileal vessels resulted in thrombosis with subsequent septic emboli to the liver via the portal system resulting in multiloculated abscesses.

Liver abscesses presenting in CD are usually multiple rather than single [4] and are frequently situated in the right lobe of the liver as demonstrated in this case. There is also clear evidence from previous cases that the most likely microorganisms to be isolated from CD related abscess are streptococcal species, in particular, S. Milleri [5]. Other species isolated include, in order of frequency, anaerobic gram-negative bacillus, and aerobic gram-negative bacillus [2].

## Figures and Tables

**Table 1:** Laboratory results including urine + stools samples.

Test	Result	Normal range
Haemoglobin	8.7 g/L	13.5–18 g/L
Mean cell volume	69 fl	80–97 fl
Platelets	575 × 10^9^/L	150–400 × 10^9^/L
White cell count	19.3 × 10^9^/L	4.0–11.0 × 10^9^/L
Neutrophills	11.48 × 10^9^/L	2.0–7.5 × 10^9^/L
Alkaline phosphate	27 u/L	30–150 u/L
Alanine aminotransferase	292 iu/L	5–35 iu/L
Bilirubin	19 *μ*mol/L	3–17 *μ*mol/L
Albumin	29 g/L	35–50 g/L
*CRP*	275 mg/L	<5 mg/L
Sodium	129 mmol/L	135–145 mg/L
Potassium	4.9 mmol/L	3.5–5.0 mmol/L
Urea	3.2 mmol/L	2.5–6.7 mmol/L
Creatinine	59 *μ*mmol/L	70–<150 *μ*mmol/L
Stool culture	Parasite negative	Culture negative
Urine sample	*Protein trace*	*Blood trace*
